# The influence of red deer space use on the distribution of *Ixodes ricinus* ticks in the landscape

**DOI:** 10.1186/s13071-016-1825-6

**Published:** 2016-10-13

**Authors:** Lars Qviller, Hildegunn Viljugrein, Leif Egil Loe, Erling L. Meisingset, Atle Mysterud

**Affiliations:** 1 Centre for Ecological and Evolutionary Synthesis (CEES), Department of Biosciences, University of Oslo, P.O. Box 1066, Blindern, NO-0316 Oslo Norway; 2 Norwegian Veterinary Institute, P.O. Box 750, Sentrum, NO-0106 Oslo Norway; 3 Department of Ecology and Natural Resource Management, Norwegian University of Life Science, P.O. Box 5003, NO-1432 Aas, Norway; 4 Department of Forestry and Forestry resources, Norwegian Institute of Bioeconomy Research, NO-6630 Tingvoll, Norway

**Keywords:** *Ixodes ricinus*, Ticks, Parasite distribution, Red deer, Large hosts, Spatial distribution, Tick management, Risk maps, Species distribution modelling (SDM), Cervid home range

## Abstract

**Background:**

Many wingless ectoparasites have a limited capacity for active movement and are therefore primarily dependent on hitchhiking on their hosts for transportation. The distribution of the tick *Ixodes ricinus* is expected to depend mainly on transportation by hosts and tick subsequent survival in areas where they drop off. In Europe, the most important hosts of adult female *I. ricinus* are cervids. The extensive space use of large hosts provides a much larger dispersal potential for *I. ricinus* than that of smaller mammalian hosts. We aim to determine the contribution of red deer (*Cervus elaphus*) space use on the spatial distribution of *I. ricinus*, after accounting for landscape factors.

**Methods:**

We analysed the spatial distribution of *I. ricinus* with generalised mixed effects models (GLMMs) based on data from extensive field surveys of questing density in two coastal regions in Norway, from which home range data from 73 red deer with GPS collars were available. Red deer home ranges were derived using the kernel method to identify areas most frequently used by deer. We first fitted a baseline model with tick questing densities relative to landscape features that are likely to affect local climate conditions and hence, survival. We then added deer space use variables to the baseline model with only landscape variables to test whether areas more frequently used by red deer had higher questing tick densities.

**Results:**

Questing *I. ricinus* density was predicted by several landscape features, such as elevation, distance to the fjord and topographic slope. In addition, we found that areas more heavily used within the red deer home ranges, correlated with higher questing tick densities. Increased effects of deer space use were additive to the landscape model, suggesting that correlations were more than just shared landscape preferences between deer and ticks.

**Conclusions:**

Our results imply that the distribution of *I. ricinus* is controlled by a complex set of factors that include both local conditions related to landscape properties that affect survival and how the large host population redistributes ticks. In particular, we have provided evidence that the local distribution of large hosts, with their extensive space use, redistributes ticks at the local scale.

## Background

The spatial distribution of ectoparasites can usually be reduced to a function of how favourable the local conditions are to their survival, the parasite's own active locomotive abilities, and passive transportation by their hosts. Different groups of ectoparasites differ largely in these traits [1]. Winged insects such as mosquitoes have high auto-locomotive dispersal potential [1], and some ectoparasites such as deer keds (*Lipoptena cervi*) have winged stages that occur prior to settling on a host and shedding the wings [2]. However, ticks have a limited dispersal potential on their own. Their small size and lack of wings make them slow to intermediate self-dispersers, and their long distance dispersal therefore depends on host movement. One of the most common ectoparasites in Europe is the sheep tick *Ixodes ricinus*, which is known to transmit several zoonotic pathogens such as *Borrelia burgdorferi* (*sensu lato*) (*s.l*.), which causes Lyme disease [3], and the virus that causes tick-borne encephalitis [4, 5]. An understanding of the factors that affect tick distribution as a function of host space use is therefore important from a public health perspective.

*Ixodes ricinus* is a three-host, three-stage hard tick (Acari: Ixodidae) that attaches to hosts and engorges continuously for a few days during every life stage [6, 7]. They spend off-host periods in the environment, either in climatically induced diapause, developmental diapause or questing for new hosts. *Ixodes ricinus* typically lives for 3–6 years [8], and the off-host period can last for many months [9]. The highest mortalities are thought to occur as a result of abiotic factors that are experienced in periods between attachment to hosts [10]. Ticks capacity for horizontal movement is limited to only a few centimetres during the most active season, and so limited horizontal movement is negligible at the landscape scale [11, 12]. Host movement and space use are therefore likely important in the distribution of *I. ricinus* [1], but few detailed studies have been conducted to explore these patterns [13].

Tick larvae and nymphs use a wide range of vertebrate host sizes, including both birds and small mammals [9, 10]. In our study area in Norway, small mammals seem to play a key role in many of these host-tick relationships [14]. The space use of rodents is often limited to a few hundred square metres and up to possibly 5,000–6,000 m^2^ [15, 16]. Adult females of *I. ricinus* can only engorge successfully on animals larger than a hare (*Lepus* spp.), and typically obtain their meal from a deer [17]. There are quite a few studies on the correlation between the density of ixodid ticks and the density of several deer species [18–20], but no study on the relationship between the distribution of *I. ricinus* and explicit measurements of deer space use based on GPS-collared animals. In Europe, roe deer (*Capreolus capreolus*) and red deer (*Cervus elaphus*) are reproduction hosts for *I. ricinus* [21], and they are the most widely distributed deer species in Europe [22]. Red deer home ranges can extend over several square kilometres [23], and we may therefore expect deer to have a considerably greater potential to affect the local distribution of ticks than do small mammals, though this remains to be documented. The role of long-distance dispersal of especially larval ticks by birds is well established [24]. However, the role of space use of large mammalian hosts for *I. ricinus* distribution at local scales has rarely been assessed.

Here, we use an existing model describing how landscape features affect the spatial distribution of *I. ricinus* to explore whether the model can be improved by adding red deer space use parameters. We term this the red deer space use-tick distribution hypothesis, and we test this hypothesis by adding red deer space use parameters to the landscape model using tick abundance data from the corresponding home ranges of red deer in the spring prior to migration and the summer after migration. We test the following predictions: (i) *I. ricinus* abundances increase towards the more frequently used centre of red deer home ranges, and (ii) *I. ricinus* abundances decrease with larger seasonal home ranges of red deer because larger home ranges have less concentrated space use.

## Methods

### Study area

**Fig. 1:**
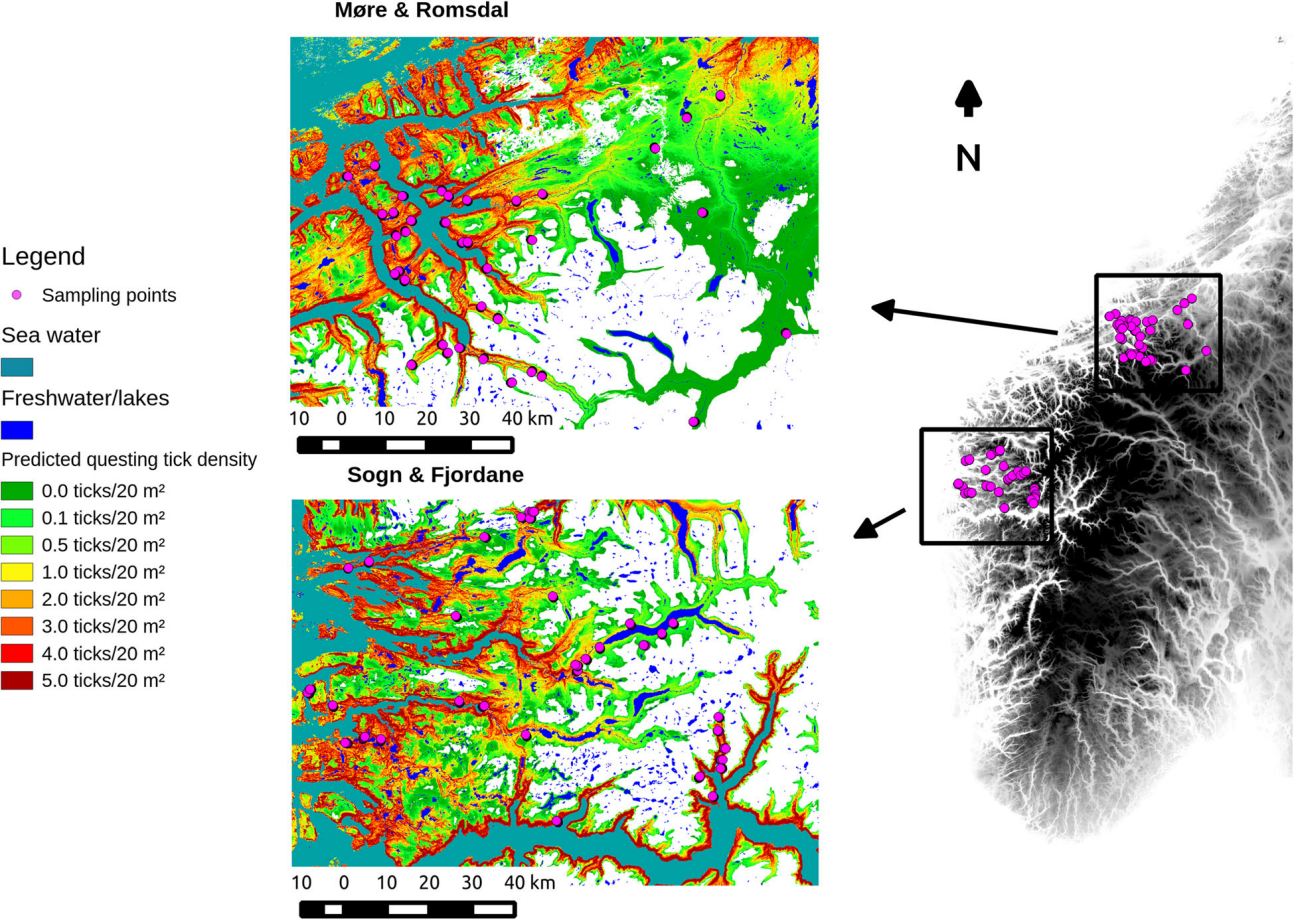
The distribution map showing the estimated questing tick densities in both study areas. The model used for predicting the distribution is presented in Table 1. The sampling transects are illustrated as *pink* dots, and predicted tick numbers collected with the flagging method in May are presented with colours, from *green* (low density) through *yellow* to *red* (high density). The map has a cut-off at 700 m a.s.l., and 68 km from the closest sea water body to avoid extrapolation outside the data range. Areas outside the data range are white. The grey map illustrates the locations of the two study areas in southern Norway

The data were collected in two areas on the west coast of Norway (Fig. 1). The first area is in the middle of Sogn & Fjordane County, delimited by the fjords Sognefjorden in the south and Nordfjord in the north (study area SF). The climate is Atlantic with mild winters and cool summers. Meteorological 30-year averages (1961–1990) for this area are 2,270 mm of precipitation and a temperature of 6.0 °C annually (http:// met.no; Norwegian meteorological station no. 57170). The second area lies in the northern parts of Møre & Romsdal County and the western parts of Sør-Trøndelag County (study area MR). This area is delimited by the Tingvollfjorden fjord in the west and the Orkdal valley in the east. Meteorological 30-year averages for study area MR is an annual temperature of 5.6 °C and 1,160 mm annual precipitation (http://met.no; Norwegian meteorological station no. 64550). The vegetation in both study areas lie within the boreonemoral vegetation zone [25]. The forests have natural stands of Scots pine (*Pinus sylvestris*), alder (*Alnus incana*) and birch (*Betula* spp.). There are also stands of Norway spruce (*Picea abies*) from extensive forest cultivation [26]. The terrain in both study areas is rugged with alpine formations. Summits and plateaus more than 1,000 m a.s.l. are common a few kilometres from the fjords. Red deer is the main deer species in the areas of SF and MR included here, though some areas have lower numbers of roe deer and moose (*Alces alces*). There are no reindeer (*Rangifer tarandus*) in these areas, as reindeer in Norway are restricted to alpine habitat. Domestic sheep (*Ovis aries*) are common in the study area. They graze in spring and late autumn mainly on fenced infields, and graze mainly on alpine habitat outside reach of most of the tick population during the summer. Mountain hare (*Lepus timidus*) is present, but in low numbers. The main surveyed areas did not have cattle (*Bos taurus*) grazing apart from two transects. Red deer are therefore the main reproduction host to ticks in the study areas.

### Red deer GPS data and space use

This study was based on GPS data from 73 adult (2 years or older) individuals of migratory and resident red deer, with 41 from Sogn & Fjordane and 32 from Møre & Romsdal County, Norway. Red deer were marked with GPS collars (Followit AB, Lindesberg, Sweden) [27–29]. The red deer were immobilised with darts at their winter feeding sites between 2005 and 2011 using a protocol approved by the Norwegian Animal Research Authority. Most collars registered the positions of the animals every hour, but some registered positions every second hour to prolong battery life. The duration of monitoring varied between a few months to 2 years. We used data only from the main tick questing period between May 1st and August 31st [30] in the present study. If there were adequate data for a particular deer for 2 years, we used the data from the year closest to the tick sampling period. Extreme outlying locations were considered to be GPS errors, and we removed them from our analyses according to standard protocols [31].

**Fig. 2:**
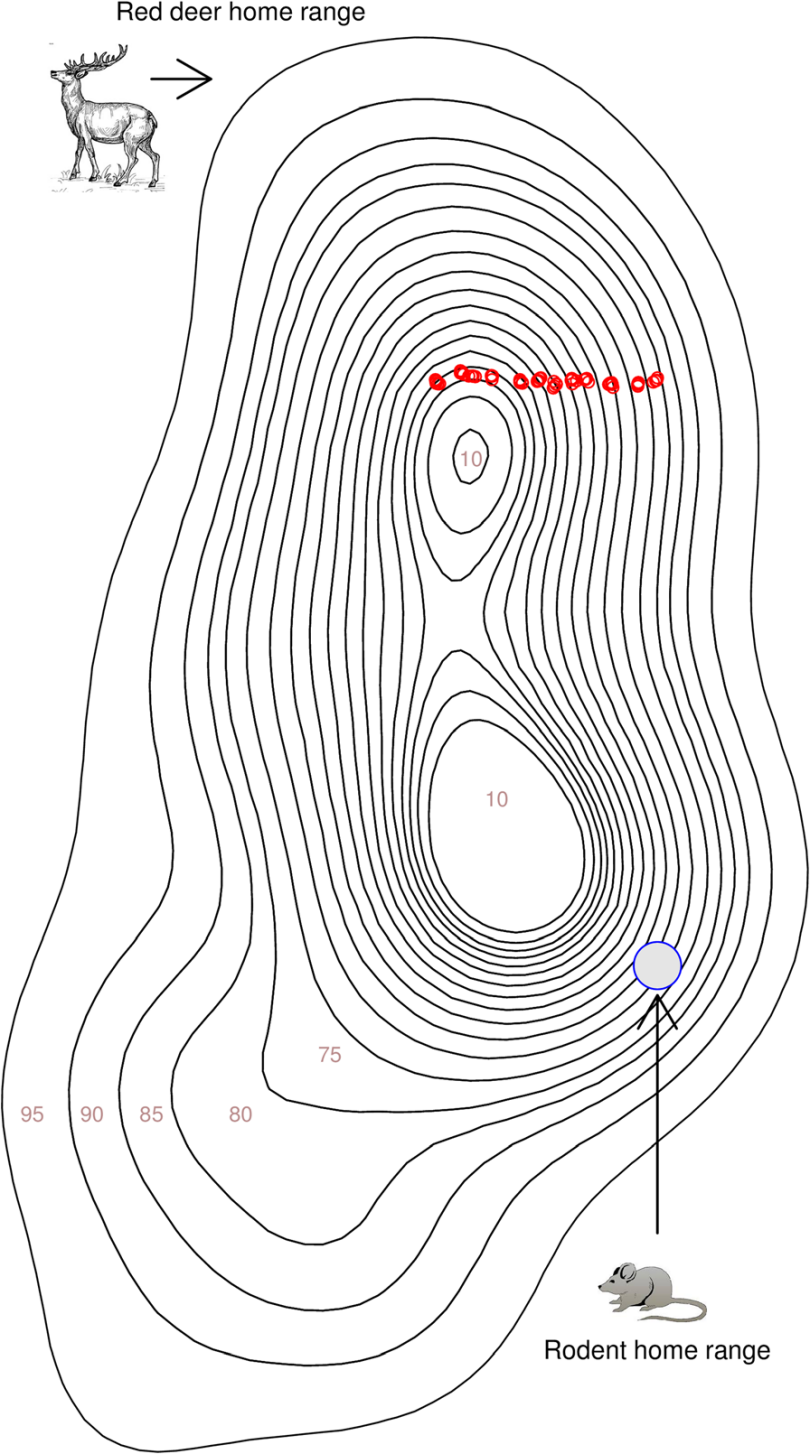
A conceptual figure of the dispersal potential for ticks that is offered by one typical red deer (95 % kernel covering 232 ha) and one typical rodent (*blue circle*). The isoclines depict kernel values for the red deer, from a 10 % kernel value in the *innermost circle*, to a 95 % kernel in the *outermost circle*, using 5 % intervals for each isocline. The data outside the 95 % isocline were given 100 % as the kernel value. The small drawing of a vole inside the 10 % kernel covers approximately one large mouse home range, ~ 0.5 ha. The *red circles* illustrate the twelve tick survey plots along one transect

We calculated the space use within red deer home ranges using the kernel methods [32] in the R-package “*adehabitat*” [33]. Kernel isoclines and isopleths define areas that account for a specified proportion of an animal’s total utilisation distribution. For example, the 50 % kernel isocline encloses the smallest area (isopleth) the monitored individual is expected to inhabit 50 % of the time, and a smaller kernel value means that the space use density is higher. We estimated 18 isopleths for each individual red deer, using percentages from 10 to 95 % in 5 % intervals (Fig. 2). Values outside the 95 % isopleths were set to 100 %. The smoothing factor (h) used was the median of the h values of all individuals, according to the reference method [32], and we examined the fit of kernel isopleths visually to validate the estimates. We divided the space use into seasonal home ranges, and kernel values were calculated separately for the spring and summer periods. The spring kernel values included GPS positions from May prior to migration for migratory individuals and for the entire month of May for resident red deer. The summer kernel values included the remainder of the summer after the spring migration event, ranging from June through August. The migration period was not included in the kernel estimates. The kernel isopleth (10–95 %) that each tick sampling plot (see below) falls within (termed “kernel”), as well as the home range size (defined as the area included within the 95 % isocline), are used as predictor variables in the analyses.

### Sampling of questing ticks

Ticks were sampled along 71 transects in May and August of the years 2009–2013 in Sogn & Fjordane and in 2011–2013 in Møre & Romsdal (resulting in one more year of data relative to [29]). Each transect consisted of twelve survey plots with randomised distances between them that were 20 to 50 m in length. The transects were established along the main elevational gradient. All transects were placed within or bordering the 95 % kernel home ranges of the GPS-marked red deer. Several animals had overlapping home ranges, and all of the summer and winter home ranges of all 73 red deer were covered by these 71 transects. In Sogn & Fjordane, we sampled 16 transects in the winter/resident home ranges and 15 transects in the summer home ranges, in addition to two transects that covered both resident and summer ranges, and one transect that covered both summer and winter home ranges (34 transects in total). In Møre & Romsdal, we sampled 20 transects in the winter/resident home ranges and 17 in the summer ranges (37 transects in total).

Ticks were collected with the flagging method [34]. The sampling equipment was a white towel (100 × 50 cm) made of cotton attached to the end of a rod as a flag, and sampling was conducted by dragging the towel over the vegetation so that questing ticks could attach. The towels were replaced if they became wet or dirty. UTM coordinates were registered using a handheld Garmin GPSmap 60CSx during every visit to the survey plots. Each of the survey plots covered approximately 20 m^2^, in a 10 × 2 m wide belt. Ticks were counted and removed after every 2 m. Tick numbers were divided into the number of nymphs, adult males and adult females for each survey plot. The tick counts for each plot are referred to as questing tick densities because the flagged area was equal in all plots. What we refer to as “tick questing density” is hence the number of adult and nymphal ticks in the 20 m^2^ survey plot. Tick questing density will reflect a combination of actual tick density as well as variation in tick activity. However, as we sample over longer time periods, we assume that variation caused by weather conditions influencing questing activity just adds noise to our data.

### Geographical covariates

Terrain data were calculated from a 10 × 10 m digital elevation raster model (DEM), retrieved from Norge Digitalt (DEM © Kartverket; http://www.statkart.no/geonorge/norge-digitalt/). We used the DEM to extract a collection of geographical properties using the GRASS GIS software [35]. The “distance to fjord” variable was calculated as the Euclidean distance to the closest sea water body, while the topographical slope and the aspect of hillsides were calculated using generic functions included in the GRASS GIS software. The output of the aspect function is degrees of east, which increases in a counter-clockwise direction. Aspect was then recalculated to the degree of northern exposure using the sinus function to avoid problems with the circular nature of this covariate. The resulting variable is termed “northness”, and ranges from -1 (southfacing) to 1 (northfacing). We also derived categories of habitat type from a land resource map provided by the Norwegian Institute of Bioeconomy Research, and the following classification was used: agricultural pastures, deciduous forests, coniferous forests, mixed forests, unclassified forests, marshes, and natural vegetation without forests. Elevation was recorded at each survey plot using GPS.

### Statistical analyses

All statistical analyses were performed using R statistical software version 3.0.2 [36]. We analysed questing tick densities with the R package “*glmmADMB*” [37]. Parasite abundance data are often overdispersed, and it is therefore common to use a negative binomial probability distribution [38, 39]. Initial analyses confirmed that negative binomially distributed errors gave the best fit, while accounting for zero inflation did not improve the model fit. We included transect as a random intercept term to account for any spatial dependency in tick density. Nonlinear relationships were explored with generalised additive models using the “*mgcv*” package in R [40]; nonlinear terms were included in the model selection procedure on the basis of this analysis. Variables were standardised (centred on the mean and divided by the standard deviation) to facilitate the comparison of effect sizes. Multicollinearity was checked with correlations and variance inflation factors as suggested by Zuur et al. [41]. It is common to separate tick instar stages in analyses of questing activity [42]. Adult ticks constitute ~10 % of the total number of ticks in these coastal areas, and previous investigations revealed no significant seasonal trends in instar compositions [29, 30]. Tick instar stages were therefore pooled prior to the analyses.

We began by fitting an overall landscape model for tick distribution in May that covered the entire study area, using the following fixed effects: study area, slope, distance to fjord, elevation as a second degree polynomial, northness and vegetation categories; transect and year were included in the model as random effects. There was no strong collinearity between any of the covariates included in the model selection procedure. All correlation inflation factors were below 2 [41]. The model is similar, but not identical, to a previously published model built using a subset of the data [29]. Relative to the published model, we added extra covariates known to affect habitat selection by red deer to ensure that any added effects of deer space use was not a result of the absence of these covariates [43].

We aimed to evaluate whether questing tick densities depend on red deer space use in addition to the landscape parameters, either in May in the home ranges that were used by red deer during winter, or in August in the ranges they used during summer. We therefore refitted the landscape model for May using only the data from the winter and resident home ranges, and for August using data only from the red deer home ranges. We then added the red deer home range parameters as fixed effects: (i) Landscape model using the questing tick densities from May in the winter/resident home ranges as the response, and then adding the winter home range variables; (ii) Landscape model using the questing tick densities from August in the summer/resident home ranges as the response, and then adding home range variables for both the resident and summer ranges of migrants.

The number of years between the sampling of ticks and the registering of red deer movements were also added to the models. This was done to control for potential changes in red deer space use and demographics during the time between deer monitoring and the quantification of tick abundance.

### Cross-validation of the landscape model

When fitting an overall landscape model for tick distribution in May, we used Bayesian information criterion (BIC) for model selection. The landscape model was evaluated using cross-validation. A reduced dataset (training data; *n* = 2718) was used to fit a training model with the specifications from the selected landscape model and to predict the test data (*n* = 500; 250 from each study area) that was not included. This procedure was repeated 400 times. Model consistency was then assessed based on the *R* ^2^-values from linear regressions between the predictions from the training model and the original landscape model. The 400 *R* ^2^ values from this analysis were presented as the median, and 5 % and 95 % quantiles of these data. For illustrative purposes, we extrapolated a model in space and presented it as a “risk map” for the predicted questing tick density in the study area in May (Fig. 1).

### Replication of individuals relative to transects

Several red deer had partly overlapping home ranges and were represented by one common transect, but most were not part of the same group and moved independently of one another. The analyses were therefore performed at the individual red deer level. Questing tick densities in May were analysed against the home range kernel values from 53 individual red deer, both migratory and resident, and these home ranges were represented by 35 of the transects in the winter/resident home range areas. Questing tick densities in August were analysed against the kernel values from resident and migratory deer in their summer/resident ranges, consisting of 70 individual red deer that were represented by 59 of the transects. This replication was handled statistically by the use of red deer identity as a random effect. However, the replication of landscape features introduces a bias towards transects with many deer individuals. This was especially a problem in the winter ranges where the overlap was more prevalent. This bias was removed using a bootstrap procedure with 400 repeated analyses. All transects that covered more than one red deer were assigned to one red deer individual at random in each of the repetitions, and estimates were calculated as the average over all 400 estimates.

This approach makes the use of likelihood-based model selection criteria more complicated. Home range is a property of the red deer identity, and it is therefore also highly correlated with the random effects. The inclusion of these parameters may therefore not improve the model likelihood, even though they explain a significant portion of the variation. Instead, the inclusion of these parameters may shift the variance explained from the random to fixed effects. The effects of the home range parameters are evaluated by whether they reduce the variance in the random effects and by their effect sizes and *P*-values.

## Results

A total number of 24,146 ticks were collected; 15,026 in May and 9,120 in summer. In May, 95 % kernel home ranges of red deer covered between 76 and 1,228 ha (median = 257), while the summer ranges covered between 84 and 2,895 ha (median = 293).

The baseline model with the landscape variables only included a positive effect of slope and a negative effect of northness (more ticks on the south face), elevation as a concave 2nd degree polynomial with a peak tick abundance at ~150 m a.s.l., and a negative effect of distance to fjord (Table 1). Pairwise comparisons showed that ticks tend to prefer forest coverage rather than marshes or areas without forest cover. The landscape model is shown as a map in Fig. 1, with a cut-off at 700 m a.s.l. and 68 km from the closest fjord to avoid extrapolation outside the data range. The model performed well when subjected to cross-validation, with a median *R* ^2^ of 0.994 (quantiles: 5 % = 0.960 and 95 % = 0.998).

**Table 1:** Parameter estimates and test statistics for the best model predicting the number of ticks caught in May with the flagging method between the years 2009 and 2013 along the west coast of Norway. Baseline is the study area in Sogn & Fjordane County and the land resource category “agricultural pastures” (intercept). Møre & Romsdal County is reported as the deviation from the baseline (Sogn & Fjordane). All model estimates are derived from standardised covariates

Parameter	Estimate	Standard error	*Z*	*P*
Intercept	-0.12	0.37	-0.33	0.73
Slope	0.34	0.040	8.4	< 0.001
Northness	-0.22	0.054	-4.2	< 0.001
Elevation	-0.28	0.094	-3.0	< 0.001
Elevation^2^	-0.28	0.070	-4.1	0.0029
Distance to fjord	-1.2	0.25	-4.5	< 0.001
Coniferous forest	-0.032	0.18	-0.18	0.86
Deciduous forest	0.29	0.17	1.7	0.08
Mixed forest	0.099	0.29	0.34	0.74
Unclassified forest	0.92	0.28	3.3	< 0.001
Marshes	-1.4	0.41	-3.4	< 0.001
Without forest	-0.47	0.29	-1.7	0.095
Study area (MR *vs* SF)	0.93	0.31	3.0	0.0030

Land resource categories have the following factor levels in addition to the intercept: coniferous forest, deciduous forest, mixed forest, unclassified forest, marshes and natural vegetation without forests (without forest)

**Fig. 3:**
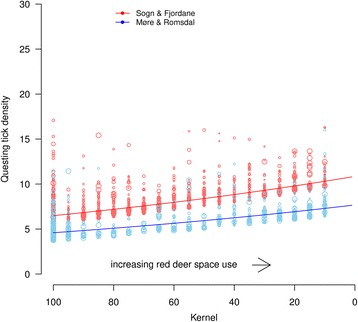
The relationship between questing tick densities and the probability of red deer space use in both study areas. Note that kernel estimates are low in areas that are used more by deer, and we have therefore inverted the x-axis to present increasing space use more intuitively as we move to the right along this axis. The data points represent the residuals from the predicted values. The sizes of the data points are scaled relative to the home range sizes, and show that higher questing tick densities occur when home range sizes decrease

The addition of red deer space use parameters from the winter/resident home ranges in May to the refitted landscape model with data from home ranges used in May resulted in a reduction in the random effect variances [variances: red deer identity = 0.88, year = 0.26 (baseline landscape only model); red deer identity = 0.73, year = 0.10 (adding deer space use)], in addition to significant *P*-values. There were no significant effects of home range parameters on the summer/resident ranges in August. As predicted, questing tick densities were negatively correlated with the home range kernel value and home range size in May, but the effect of home range size was not significant (*P* = 0.061; Table 2, Fig. 3). These results indicate that increases in the amount of time spent in an area by a red deer increases the number of ticks. The tracking of deer with GPS and the survey for ticks was not done the same year. In addition, there was a negative correlation between tick abundance and the number of years between deer monitoring and tick sampling, i.e. there were fewer ticks when there were more years between red deer monitoring and the tick survey.

**Table 2:** Parameter estimates for the landscape model that also includes kernel estimates from red deer home ranges. The model predicts the numbers of ticks caught in May, representing winter and resident home ranges, with the flagging method between the years 2009 and 2013. Baseline is the study area in Sogn & Fjordane County (intercept) and the land resource category “agricultural pastures”. Møre & Romsdal County is reported as the deviation from the baseline (Sogn & Fjordane). Estimates are averaged over all 400 estimates from the bootstrap analysis

Parameter	Estimate	Standard error	*Z*	*P*
Intercept	2.2	0.51	4.3	< 0.001
Northness	-0.21	0.050	-4.3	< 0.001
Slope	0.36	0.046	7.8	< 0.001
Elevation	0.015	0.076	0.20	0.73
Elevation^2^	-0.079	0.051	-1.6	0.14
Distance to fjord	-1.1	0.17	-6.6	< 0.001
Coniferous forest	0.067	0.17	0.40	0.63
Deciduous forest	0.49	0.16	3.0	0.020
Mixed forest	0.53	0.35	1.5	0.15
Unclassified forest	1.1	0.27	3.9	< 0.001
Marshes	-1.3	0.49	-2.6	0.016
Without forest	-0.043	0.26	-0.17	0.66
Kernel	-0.21	0.066	-3.2	0.012
Years between	-0.30	0.074	-4.1	< 0.001
Home range size	-0.15	0.072	-2.1	0.062
Study area (MR vs SF)	-0.50	0.45	-1.1	0.27

Land resource categories have the following factor levels in addition to the intercept: coniferous forest, deciduous forest, mixed forest, unclassified forest, marshes and natural vegetation without forests (without forest). Estimates are derived from standardised covariates. Note that this is a subset of data used in Table 1. Note also that high kernel values indicate that an area is used less frequently by red deer

## Discussion

Here, we provide evidence that the density of questing *I. ricinus* ticks is higher in the core than in the periphery of individual red deer home ranges. The improved explanatory power of the addition of red deer space use metrics to a baseline landscape model indicates that the effect of red deer space use was a result of more than just shared landscape preferences of ticks and deer. This suggests that fine-scale variation in the space use of large hosts affects the distribution of ticks in this northern forest ecosystem.

Tick distributions are known to follow climatic gradients. The baseline landscape models include factors that correlate with the climatic conditions to avoid that these factors cause bias. That large hosts such as deer can also have an impact on the distribution of *I. ricinus* is an established concept [17, 44]. It has been suggested that these large hosts may act as vehicles that distribute these parasites in the landscape [13]. We found that the individual home range kernel value (i.e. how much time the deer spent within a given area) and the time lag between red deer monitoring and tick sampling were significant predictors of questing tick density, but only in May. As predicted from the red deer space use-tick distribution hypothesis, the correlations between tick density and both home range kernel values were negative. This means that tick density increased with the amount of time a red deer spent in an area (Table 2, Fig. 3), thus supporting the hypothesis that red deer transport ticks in the landscape. The home range kernel value is scaled relative to an individual deer’s space use and is therefore calculated independent of large scale deer densities. It may still act as a proxy for more general habitat selection of red deer in that area and is thus also a proxy for local deer density. The home range size of deer in general may decrease with increased densities (e.g. for roe deer [45]). The effect of home range size on tick abundance was negative, but marginally non-significant. The size of a home range reflects how concentrated the area is being used by red deer individuals, the local red deer population density and the overlap with the core home ranges of other unmarked individuals. In principle, such co-occurrence may also be due to a common preferred, but unmeasured habitat variable [46]. The negative effect of the time lag between deer monitoring and the tick sampling adds further evidence to an association between the GPS-marked red deer (and associated individuals) and tick distribution, as the landscape variables remain constant while the effect of an absent red deer gradually decreases away over time.

We found no association between red deer space use during summer and the tick density in August. There is great variation in questing activity throughout the active season [30]. Ticks in diapause and ticks engorging on hosts are not detected with the flagging method and are therefore difficult to estimate [10]. It is possible that the increased pick-up rate by hosts counteracts the effect of deer space use as the summer progresses, thus reducing the effect of red deer space use on the density of remaining questing ticks in August. Additionally, the social organisation of red deer changes from spring to summer. During spring, the red deer often remain in larger herds, so the space use of a particular GPS-marked individual may represent a large number of deer. During summer, red deer spread out and females live in small family groups of 2–3 individuals [47].

Rodents have very limited home range sizes compared to deer species (Fig. 2). Bank voles (*Myodes glareolus*), for example, typically use between a few hundred square metres and 0.5 ha (0.2 ha on average) [15, 16]. One half hectare is the size of a circle with an approximately 40 m radius, which is an area comparable to that of a large garden. Such movements have little effect on the distribution of ticks at a larger scale [48]. In contrast, the red deer in the present study have a large dispersal potential for ticks, as they use large areas that range between 78 and 1,228 ha during May (Fig. 2). If a tick drops off its host and falls to the ground, it will likely remain within or near the same area until it is picked up again by a host. Hence, the effect of deer space use, and kernel estimates in particular, both reflect the limited movements of ticks and the space use by abundant smaller hosts. Birds also have the potential to transport ticks over large distances [24, 49, 50]. A study of the biogeography of Lyme disease spirochetes in Europe have demonstrated that the genetic structures of different *Borrelia* genospecies were linked to the movement capabilities of their vertebrate hosts [51]. *Borrelia afzelii,* which specialises on rodents, had a highly genetically structured distribution, while *B. garinii,* which specialises on avian hosts, showed evidence of greater genetic mixing [51]. However, the genospecies composition of *B. burgdorferi* (*s.l*.) in Norway is dominated by the small mammal specialist *B. afzelii*, providing evidence that the most common hosts for immature ticks are rodents [52]. It is therefore likely that the genetic structure of ticks follows red deer movement patterns in this northern ecosystem.

## Conclusion

Ticks have a limited capacity for locomotion, and their distribution in the landscape is therefore assumed to follow the movements of their hosts. We have provided evidence that the variation in space use by red deer correlates with the local distribution of ticks on the western coast of Norway. Our study highlights that the local distribution of large hosts, with their extensive space use, redistributes ticks at the local scale.

## Abbreviations

AIC: Akaike information criterion
BIC: Bayesian information criterion
DEM: Digital elevation raster model
GLMMs: Generalised mixed effects models
GPS: Global Positioning System
*s.l*.: *sensu lato*
UTM: Universal Transverse Mercator.
